# Association Between Cognitive Function and Spatial–Temporal Measures of Gait and Balance When Navigating a Virtual Reality Floor Maze

**DOI:** 10.1109/TNSRE.2025.3605536

**Published:** 2025

**Authors:** Jiawei Chen, Dario Martelli, Sunil K. Agrawal

**Affiliations:** Department of Mechanical Engineering, Columbia University, New York, NY 10027 USA; Orthopedics/Sports Medicine Department, MedStar Health Research Institute, Baltimore, MD 21218 USA; Department of Mechanical Engineering and the Department of Rehabilitation and Regenerative Medicine, Columbia University, New York, NY 10027 USA

**Keywords:** Floor maze test, spatial navigation, cognition, gait and balance, virtual reality

## Abstract

Spatial navigation has been used as a behavioral marker of cognitive impairments. Floor Maze Tests (FMT) are used to characterize navigation where subjects physically move through a two-dimensional maze drawn on the floor. A Virtual Reality version of FMT (VR-FMT) has been developed, which provides a 3-dimensional navigation environment where the height of the maze walls can be altered. For both FMT and VR-FMT, the time used to complete the maze has been reported as the outcome measure to characterize the cognitive function. This study aims to show new performance metrics derived from spatial-temporal gait and balance parameters during navigation through the maze and their association with the cognitive scores in subjects with no dementia. Sixty-five older adults with no dementia participated in an experiment where subjects walked in VR-FMT with two wall heights, 2 centimeters (no wall condition) and 2 meters (wall condition). Our results showed that in no wall condition, the gait and balance parameters during navigation were associated with cognitive scores measuring attention and executive function. In wall condition, besides attention and executive function, gait parameters showed a correlation with the scores of the auditory memory. This paper showed that the spatial-temporal gait and balance parameters during spatial navigation are important metrics of cognitive function in addition to the completion time. VR-FMT with walls can help identify early memory impairments in individuals.

## I. Introduction

SPATIAL navigation refers to the process of determining and traversing a route from one point to another [1]. Spatial navigation deficits are seen in early clinical stages of Alzheimer’s disease (AD) and have been reported in predementia syndromes, such as mild cognitive impairment syndrome (MCI) [2], [3], [4]. There is emerging interest in characterizing outcomes of spatial navigation and how these depend on participants’ cognitive scores [5].

The Floor Maze Test (FMT) has been employed to investigate navigational ability [6], [7]. In this test, subjects walk through a 2-dimensional maze drawn on the floor. The participants are first instructed to observe the maze during a planning period and remember a path leading to the exit point of the maze. In the execution phase, they physically walk through the maze. Navigational ability has also been evaluated with pencil and paper, where participants draw a path with pencil from a start point to an exit point of a maze or computer-based assessment where participants navigate using a hand-held controller [8]. In comparison to more traditional pencil and paper tests, physical navigation in Floor Maze Tests additionally requires the integration of sensory signals from vestibular, proprioceptive, and podokinetic feedback [9] and is better able to identify subjects with cognitive impairments [10].

In FMT, the time of completion of the maze has been used as the primary performance metric [6]. However, completion time is an aggregate measure of maze navigation and is typically influenced by the walking speed of the person and the stochastic nature of the events, including making wrong turns within the maze.

Spatial-temporal gait and balance parameters are important characteristics of walking that differentiate gait of healthy subjects from those with impairments. Research suggests that gait and balance deficits with coexisting cognitive impairment can be a strong risk factor for dementia [11]. During maze navigation, the spatial-temporal gait and balance parameters will likely change during walking when a subject has to divide attention between planning a path and executing it [12]. The adaptations in gait and balance during maze navigation can be an important metric to characterize individuals as they switch between the cognitive task of planning the path and the motor task of executing it.

In FMT, subjects may be able to visualize the whole maze from a single vantage point and use distal visual clues to plan and execute the path [2]. The Virtual Reality version of the Floor Maze Test (VR-FMT) has been proposed, which can provide a realistic maze in virtual reality while also altering the complexity of the maze or adding height of the walls in the maze during navigation [13]. In the VR-FMT with walls, the subjects need to localize their current position in the maze and rely on memory during navigation [14]. The previous study indicates that completion times in the VR-FMT with walls correlate with subject scores in attention and executive function [13]. However, other performance outcomes, including spatial-temporal parameters, were not investigated. Additionally, the study did not examine correlations between VR-FMT measures and other cognitive tests, such as those assessing memory, verbal abilities, and visuospatial skills.

In this paper, we conducted a study with older subjects to examine their gait and balance while traversing a maze and correlations with their cognitive test scores. The study also assesses how these associations were influenced by the presence of walls. Subjects navigated through a VR-FMT under two conditions: a no-wall condition (wall height set to 2 centimeters) and a wall condition (wall height set to 2 meters). We calculated the spatiotemporal gait parameters, including stride length, stride width, stride time, stride velocity, stance time percentage, and their variability during navigating in the VR-FMT [15]. Changes in balance are represented by changes in the mediolateral margin of stability and the mediolateral center of mass displacement [16], [17]. Participants performed standard cognitive tests that characterize their attention, executive function, verbal fluency, visual and auditory memory, and visuospatial reasoning.

We hypothesized that (i) gait and balance parameters during navigation would be correlated with cognitive scores. (ii) In addition to attention and executive function, performance outcomes in a VR-FMT with walls would be significantly correlated with cognitive tests in the memory domain.

## II. Method

### A. Study Population

Sixty-five subjects (16 male and 49 female, age: 67.8 ± 6.3 years, height: 167.7 ± 8.6 cm, weight: 77.4 ± 17.7 kg, education 16.9 ± 3.2, mean ± SD) participated in this study. Subject inclusion criteria were: (i) age ≥ 60 years; (ii) able to safely walk without mobility aids; (iii) corrected vision of at least 20/40; and (iv) able to understand and read English. General exclusion criteria were: (i) self-reported medical conditions or any history of neurological disease (e.g., stroke, Parkinson’s, Alzheimer’s), severe traumatic head or drug and substance abuse; (ii) acute, severe, or unstable medical illness; (iii) having experienced a lower limb muscle injury in the 3 months before the study; (iv) having undergone surgery in the year before the study; (v) having probable dementia (scoring below 20 - or 21 for high school education - in the St. Louis Mental Status Examination); and (vi) being at higher risk for falls (scoring below 8 in Short Physical Performance Battery [18]). Study procedures were approved by the Institutional Review Board at The University of Alabama (IRB: 21-07-4777). The IRB approval date was September 8, 2021.

### B. Cognitive Assessment

Research assistants administered an extensive neuropsychological battery of tests to each participant in the study. Specific cognitive tests were given to the subject that targeted attention (Trail Making Test Part A [19]), executive function (Digit Symbol Substitution Test, Trail Making Test Part B [19], and Digit Comparison Test), verbal fluency (Control Oral Association Test - ‘F’, ‘A’ and ‘S’ [20], Word Reading Subset from Wide Range Achievement Test-Fourth Edition [WRAT4] [21]), auditory memory (Logical Memory Test from the Wechsler Memory Scale Edition IV [WMS-IV] [22]), and visual memory (Designs Test from the Wechsler Memory Scale Edition IV [WMS-IV] [23]). For the Logical Memory Test and the Designs Test, in addition to the initial assessment, which is labeled as ‘immediate test’, the same tests were administered again after a 10-minute delay, which we labeled as ‘delayed test’. Additional testing of visuospatial reasoning included a paper-and-pencil Porteus Maze Test [8].

### C. Virtual Reality Floor Maze

The virtual reality floor maze environment was created using Unity3D software (Unity Technologies, San Francisco, CA, USA) and projected in first-person view using a wireless head-mounted VR headset (VIVE HTC PRO, Valve Corporation, Bellevue, WA, USA).

**Fig. 1.**
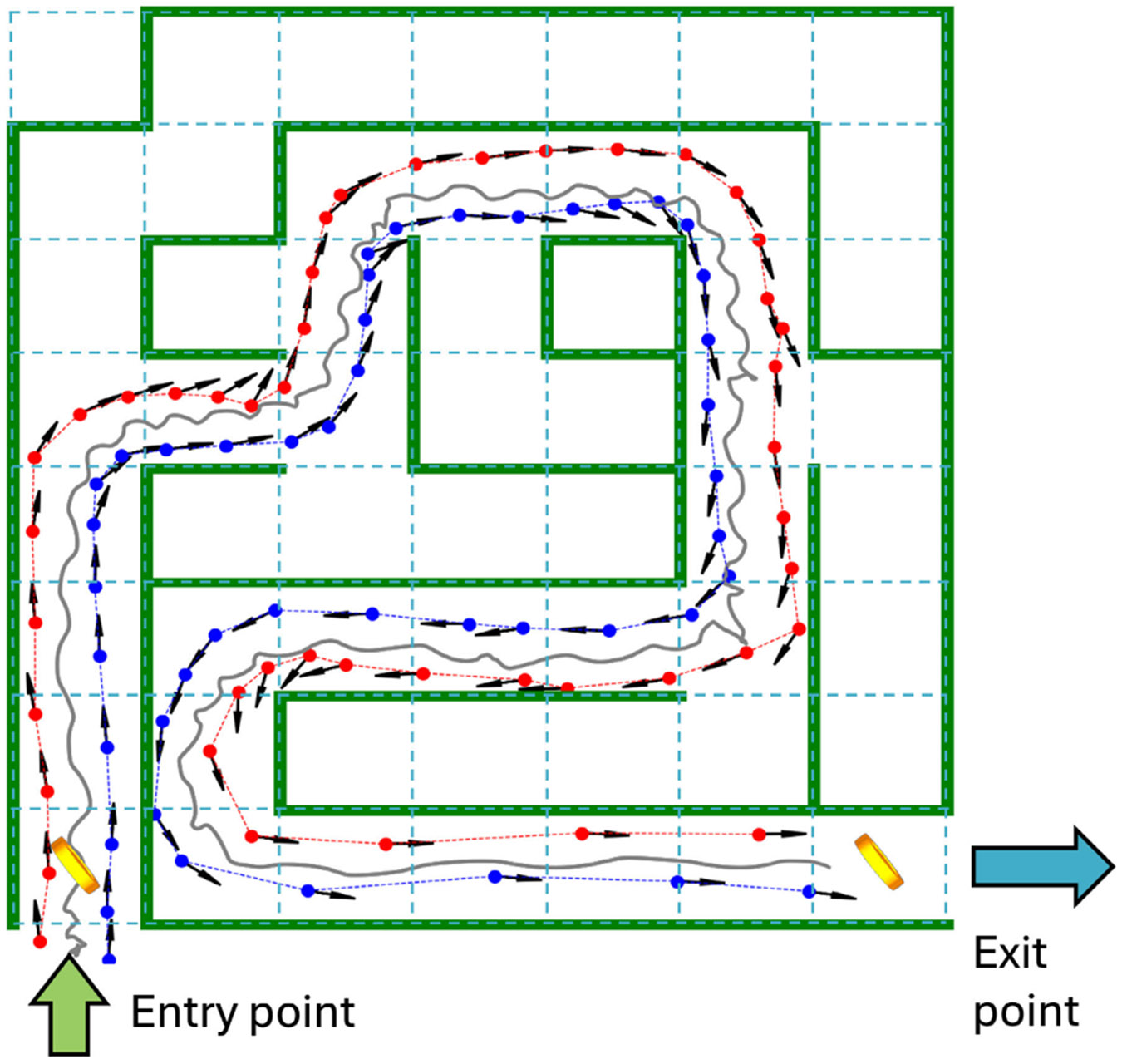
Example of a path taken by a participant in a virtual reality floor maze. Two coins are displayed at the entry and exit points of the maze. A path taken by a subject during navigation is shown in the plot. The gray path represents the pelvis movement during navigation. Red and blue dots represent the positions of left and right heel strikes detected using the trackers on the foot. The black arrow represents the foot direction at the heel strikes.

Fig. 1 shows a virtual reality floor maze with a representative path for a subject from the entry of the maze to the exit. A virtual reality floor maze is constructed with a block template [13], each measuring 0.56 m × 0.56 m. Each maze consists of 56 blocks arranged in a 7 × 8 grid. All mazes have an entry point at a corner and the navigation path from the entry point to the exit point of the maze is unique.

### D. Experimental Protocol

Four virtual reality floor mazes of similar complexity were preprogrammed for this experiment. For each subject, two different mazes were randomly selected from the four preprogrammed mazes, one maze was used in the no-wall condition and the other was used in the wall condition.

**Fig. 2.**
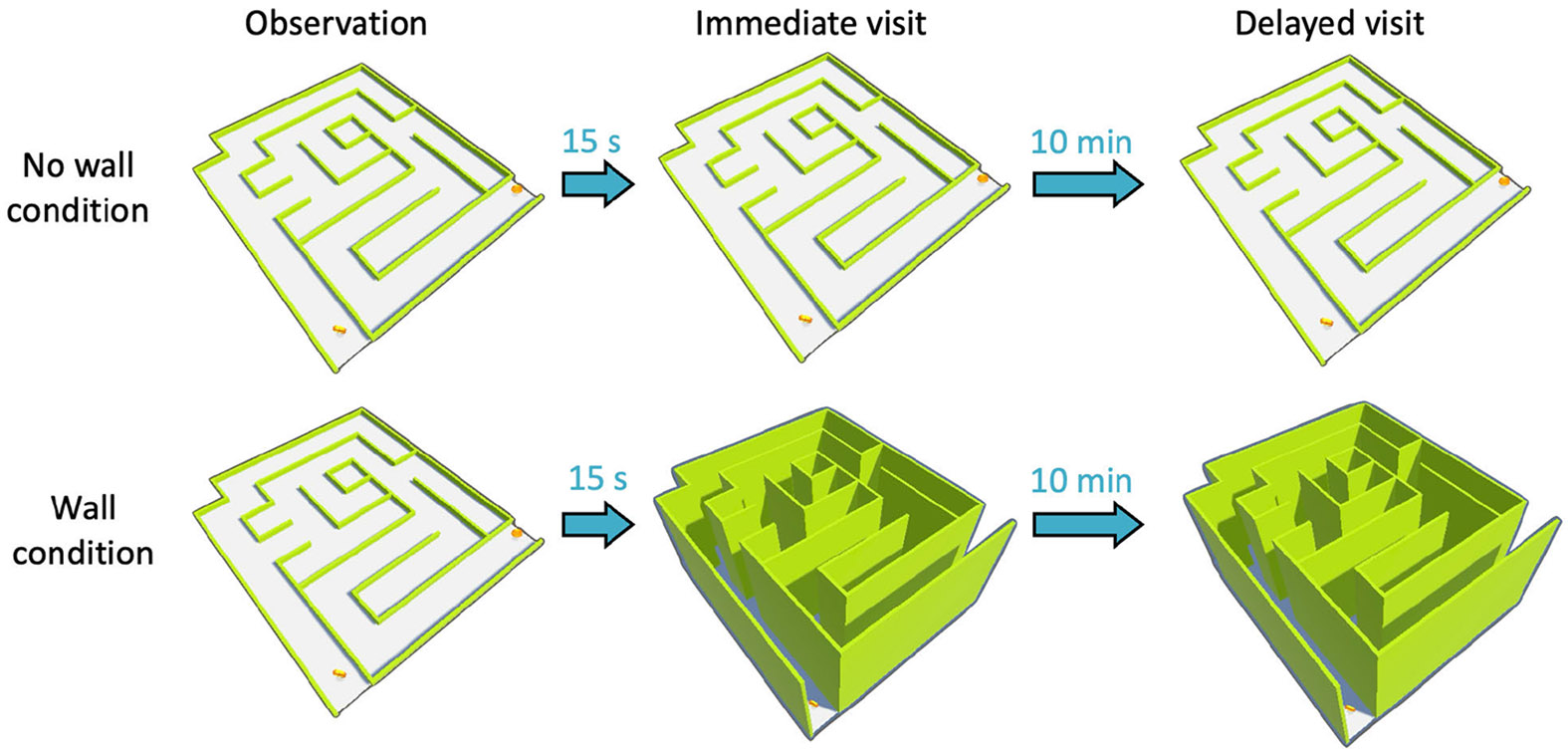
Experiment setup. (a) Experiment protocol. In both conditions, subjects observe the maze with no-walls for 15 seconds before navigating through the maze. Two coins are shown at the entry and exit point of the maze. After observing the maze, subjects navigate through the maze with No-Wall and Wall conditions, respectively (immediate visit). The subjects then took a 10-minute rest and repeated navigating through the maze (delayed visit).

Subjects completed the VR-FMT in both no-wall and wall conditions. The experiment protocol is shown in Fig. 2a. In both conditions, a research assistant positioned the subject at the entry point of the maze and instructed them to plan the path to the exit. A fixed 15-second planning period was given to each subject. Following the planning phase, the subject was instructed to walk towards the exit point, which we labeled as ‘immediate visit’. Then each subject repeated the same maze after a delay of 10 minutes with no planning period allowed. We labeled this as ‘delayed visit’. For both immediate and delayed visits, subjects were given as much time as they needed to walk to the exit of the maze. In the wall condition, once the planning phase was completed, the wall was raised to a height of 2 meters. The subject repeated the maze with the wall raised after rest. The order of the no-wall and wall conditions was randomized among subjects.

Before the experiment, three 6-degrees-of-freedom position and orientation trackers (HTC VIVE Tracker 2.0) were placed on the subject, as shown in Fig. 2b. One tracker was placed on each ankle with the tracker’s x-axis pointing along the foot. The third tracker was placed on the navel of the subject with the x-axis pointing upward. Tracker position and orientation data were collected at a 90 Hz sampling rate.

### E. Gait Events Detection

**Fig. 3.**
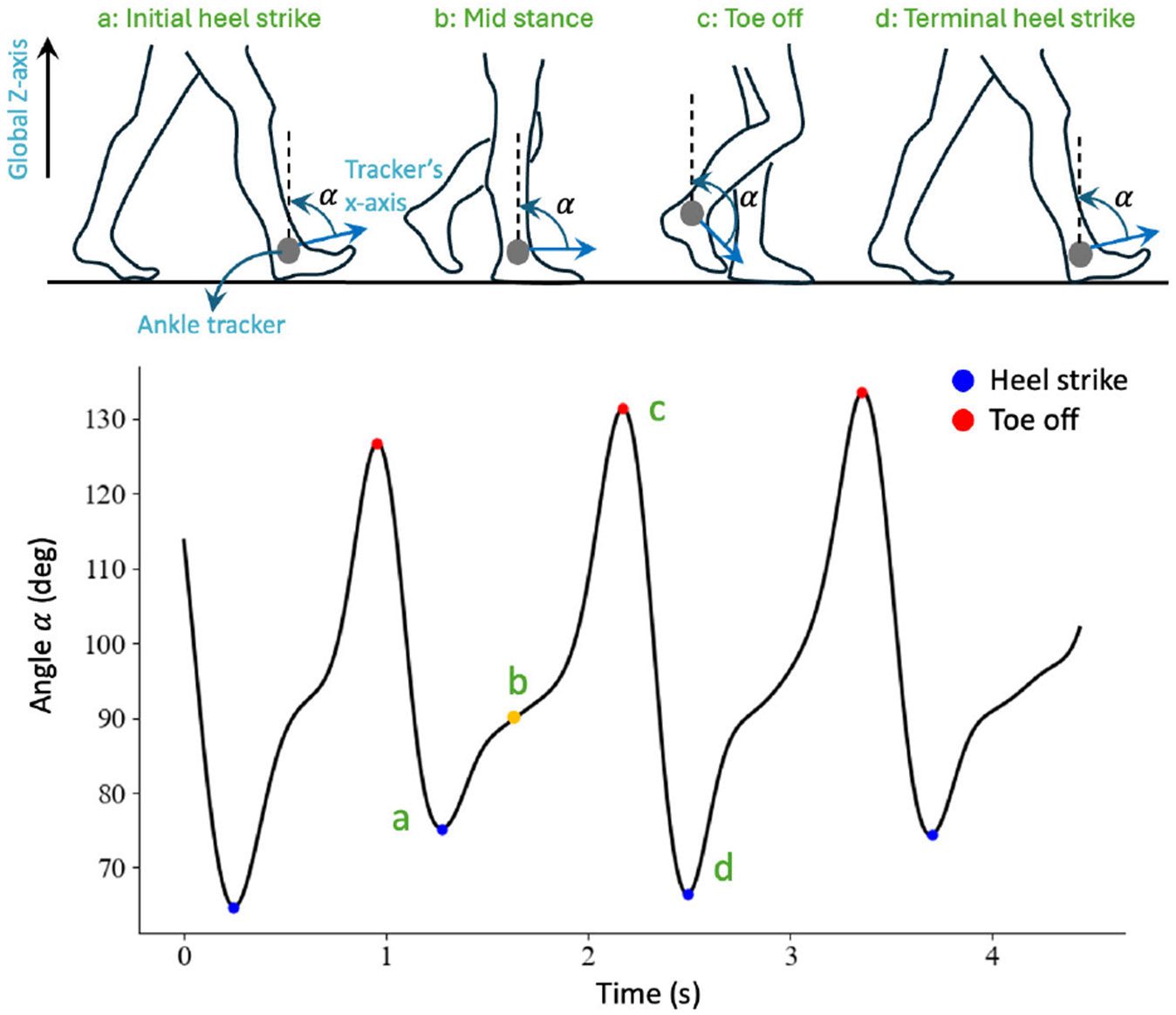
Top: schematic of foot movement in a gait cycle. Bottom: angle *α* between tracker’s x-axis and fixed global Z-axis in three consecutive gait cycles. Within a gait cycle, the angle *α* increases from initial heel strike to toe-off (marked points a and c) and decreases from toe-off to terminal heel strike (marked points c and d). The heel strike events were defined as the local minima of the waveform. The toe-off events were defined as the local maxima of the waveform.

We analyzed the data from the ankle trackers to determine the gait events, as shown in Fig. 3. Heel strike was defined when the angle between the tracker’s local x-axis and global Z-axis reached a local minimum. Toe-off was defined when the angle between the tracker’s x-axis and the world Z-axis reached a local maximum value [24].

### F. Gait Parameters

Steps in maze navigation were classified into straight and turning steps as a maze has frequent right and left turns [25]. In this paper, we classify straight and turning steps using the foot direction, which was defined by the projection of the tracker’s x-axis on the horizontal plane. A step is classified as a straight step if the deviation in foot direction between two consecutive heel strikes remains below a manually selected threshold, which varies from 20 to 30 degrees. This threshold is specifically determined by the foot direction change during straight line walking in the maze. Steps exceeding this threshold are designated as turn steps [26].

Only straight steps were analyzed in this study as the number of turning steps was limited. Spatiotemporal gait parameters such as stride length, stride width, stride time, stride velocity, and stance time percentage were computed using position and orientation data from VR trackers [26]. The coefficient of variation (CoV) of a variable was defined as the quotient of the standard deviation and its mean value [27]. This was calculated for each variable.

### G. Balance Parameters

The extrapolated center of mass (XCoM) is defined as:

(1) $$\vec{\text{XCoM}}=\vec{u}+\frac{\dot{\vec{u}}}{\omega_{0}}\;\;\text{and}\;\;\;\omega_{0}=\sqrt{\frac{g}{l}}$$

where $\vec{u}$ is the center of mass (CoM) position, approximated by the tracker positioned at the navel [28], $\dot{\vec{u}}$ is the CoM velocity and g is the gravitational acceleration (9.8 m/s^2^). l is the leg length defined as the vertical distance between the foot tracker and the navel tracker [28].

**Fig. 4.**
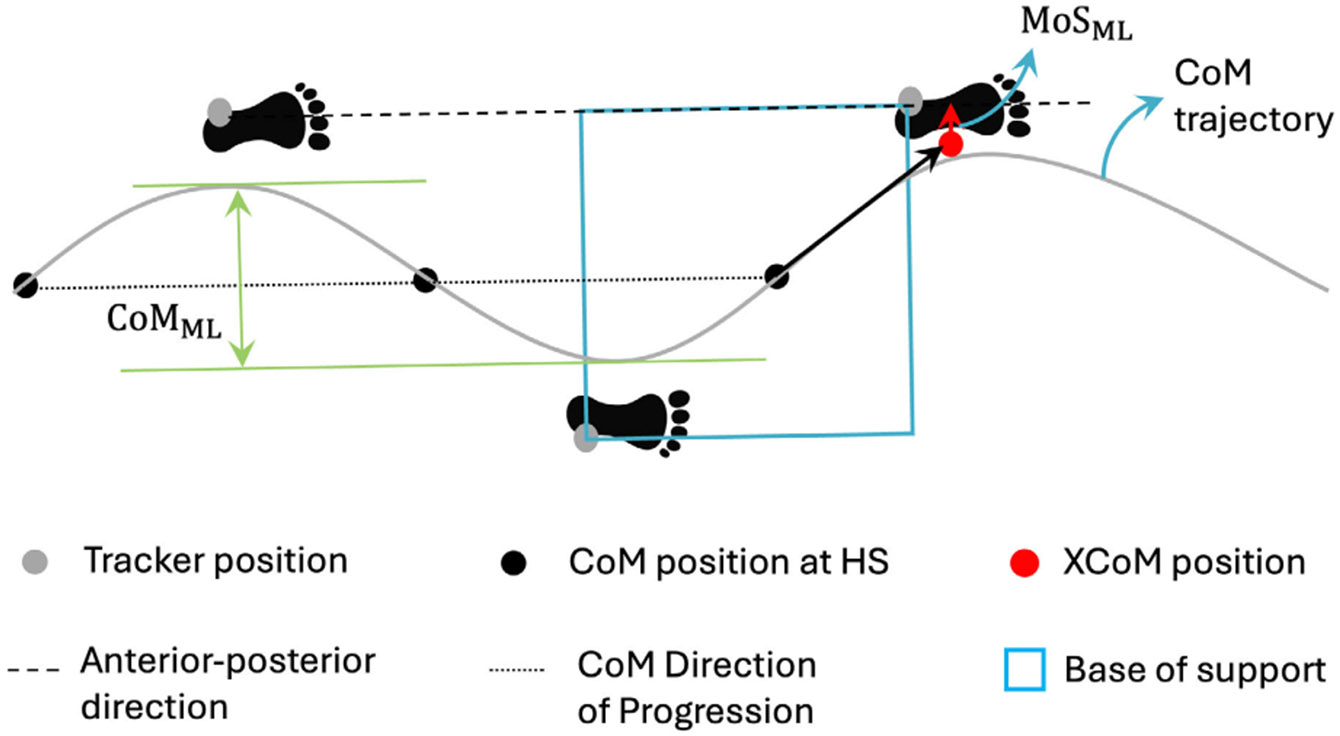
Schematic of mediolateral margin of stability (MoS_ml_) and mediolateral center of mass displacement (CoM_ml_) calculation. Black dots represent the center of mass (CoM) positions at the heel strikes (HS).

The anterior-posterior direction for the step was defined by the vector pointing from the initial to the terminal heel strike in that gait cycle. The mediolateral direction was taken as perpendicular to this vector. The base of support (BoS) was defined as a rectangle covering the feet, aligned with their major dimensions. The borders of this rectangle were determined by the trackers placed on the ankle. The margin of stability (MoS) is defined as the minimum distance between the XCoM and BoS [16]. The calculation of the mediolateral margin of stability MoS_ml_ is as shown in Fig. 4. In this study, we calculated MoS along the mediolateral direction at every heel strike [29].

In a gait cycle, the CoM direction of progression (DoP) was defined as the vector pointing from the CoM position at the initial heel strike to the CoM position at the terminal heel strike in that gait cycle. The CoM mediolateral displacement CoM_ml_ was calculated as the maximum deviation of the CoM relative to the DoP [30]. The CoV for the CoM mediolateral displacement was also calculated.

### H. Statistical Analysis

Statistical significance was set at *p* < 0.05. Data normality tests were performed on the spatial-temporal gait parameters and their coefficients of variation by the Shapiro-Wilk test and visually inspected with Q-Q plots. Non-normally distributed data were transformed using the logarithmic transformation.

During the experiment, the tracker experienced loss of tracking in a few conditions due to battery issues and occlusion. Among the 65 subjects, at least one tracker lost tracking for ten subjects in one condition, for three subjects in two conditions, for another three subjects in three conditions, and for one subject in all four conditions.

We used multivariable linear regression analyses to examine the association of neuropsychological test results with outcome measures of the maze navigation including completion time, spatiotemporal gait and balance parameters. Regression models were adjusted for potential confounders including age, sex, education, height, and weight.

To accommodate missing data, we employed linear mixed-effects models to determine the main and interaction effects of the conditions (No-wall and Wall) and repetitions (Immediate and Delayed visits) on gait and balance parameters [31]. Completion time, gait, and balance parameters were used as dependent variables. The linear mixed models included a two-way interaction term between condition and time and accounted for the subject as a random effect. Significant interaction effects were followed up by pairwise comparisons with Bonferroni adjustment.

## III. Results

A list of neuropsychological tests and their performance by subjects is summarized in Table I.

### A. Regression Analysis

**Fig. 5.**
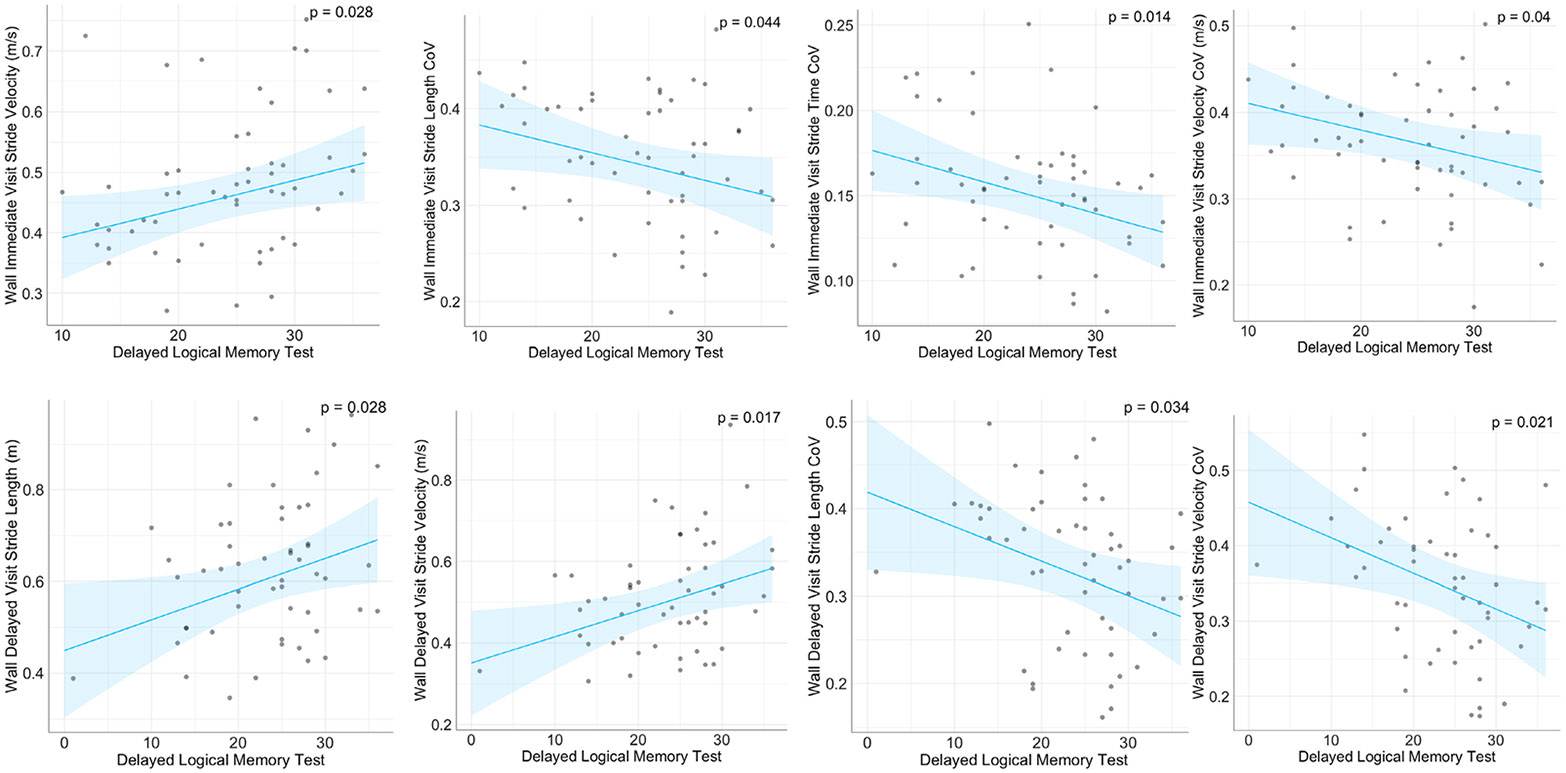
Regression plots for variables significantly associated with tests in the memory domain. P values of the regression analysis are indicated and adjusted for demographic variables including age, gender, height weight, and education. The blue area reflects the 95% confidence level interval for predictions of the linear regression model.

Selective regression analysis results are reported in Table II, variables are presented if they are significantly associated with at least one neuropsychological test. For the Wall condition immediate visit, the increased scores from the Delayed Logical Memory Test were associated with increased stride velocity, and reductions in the coefficients of variation (CoV) for stride length, stride time, and stride velocity. For the Wall condition delayed visit, the increased scores from the Delayed Memory Test were associated with increases in both stride length and stride velocity, as well as decreased CoVs for stride length and stride velocity. Fig. 5 reports the scatter plots of the gait variables significantly associated with the Delayed Logical Memory Test.

### B. Effects of Walls on Spatial-Temporal Gait and Balance Parameters

#### 1) Completion Time:

In the no-wall condition, completion times (mean ± SD) were 28.4 ± 10.9 sec for the immediate visit and 26.1 ± 9.43 sec for the delayed visit. In the wall condition, completion times were 64.3±46.7 sec for the immediate and 49.7 ± 33.6 sec for the delayed visit. No interaction effect between condition (no-wall and wall) and repetition (immediate and delayed visits) was observed for completion time. Completion time in the wall condition was significantly higher than in the no-wall conditions (*p* < 0.001).

#### 2) Gait Parameters:

**Fig. 6.**
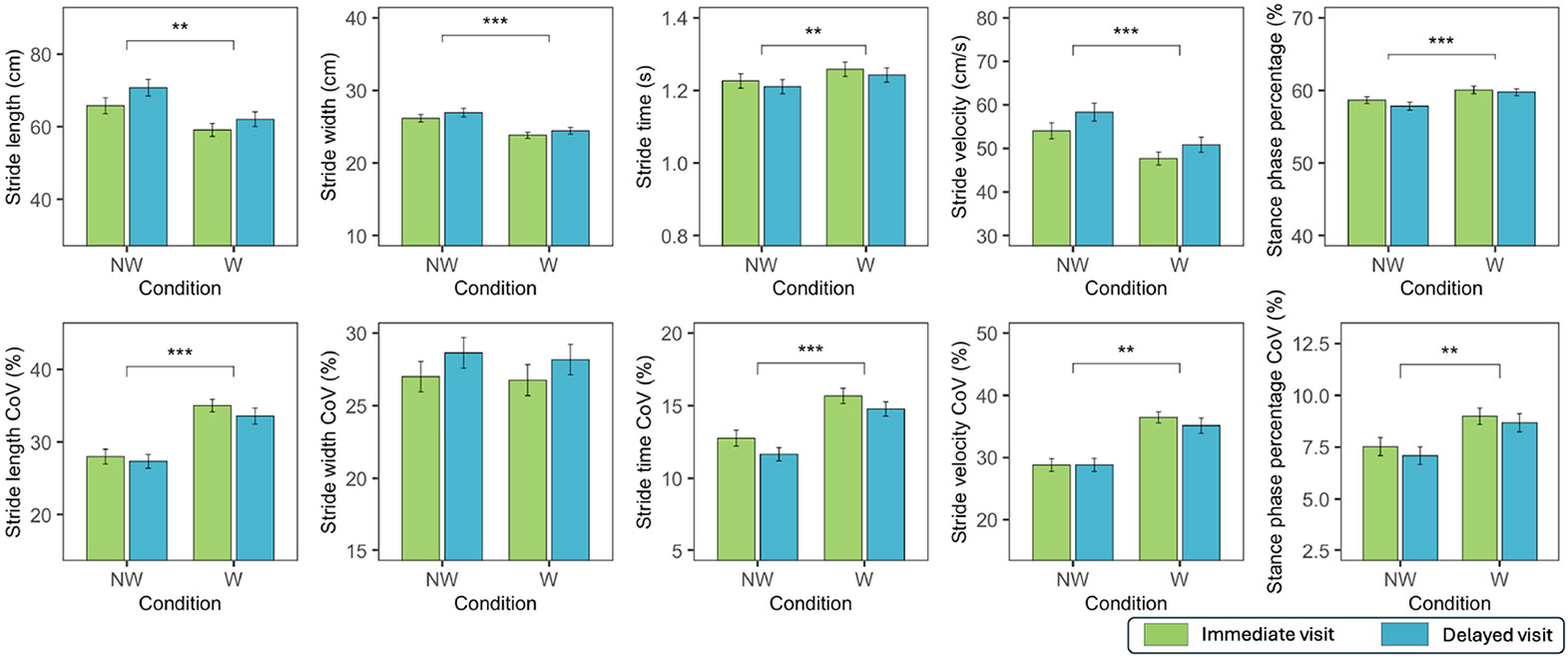
Gait parameters and their coefficient of variation (CoV) in virtual reality floor maze in no-wall (NW) and wall (W) conditions in the immediate and delayed visit. * symbols indicate a significant difference between conditions (* = *p* < 0.05, ** = *p* < 0.01, * * * = *p* < 0.001). Values are reported as mean ± one standard error.

Fig. 6 shows the gait parameters in no-wall (NW) and wall (W) conditions for both the immediate and delayed visits. No interactions were observed for these variables and their variability, meaning the relationship of variables between no-wall and wall conditions did not change across the immediate and delayed runs.

Stride length was significantly lower in wall condition (*p* = 0.003) and increased significantly from the immediate visit to the delayed visit (*p* < 0.001). Stride width was significantly lower in wall condition (*p* < 0.001). Stride time was significantly higher in wall condition (*p* = 0.006). Stride velocity was significantly lower in wall condition (*p* < 0.001) and increased in the delayed visit (*p* < 0.001). Stance phase percentage was significantly higher in wall condition (*p* < 0.001).

Stride length variability was significantly higher in the wall condition (*p* < 0.001). Stride width variability was significantly lower in delayed visit (*p* = 0.003). Stride time variability was significantly higher in wall condition (*p* < 0.001). Stride velocity variability was significantly lower in wall condition (*p* < 0.001) and increased in the delayed visits ( *p* = 0.002). Stance phase percentage variability was significantly higher in wall condition (*p* = 0.001).

#### 3) Balance Parameters:

**Fig. 7.**
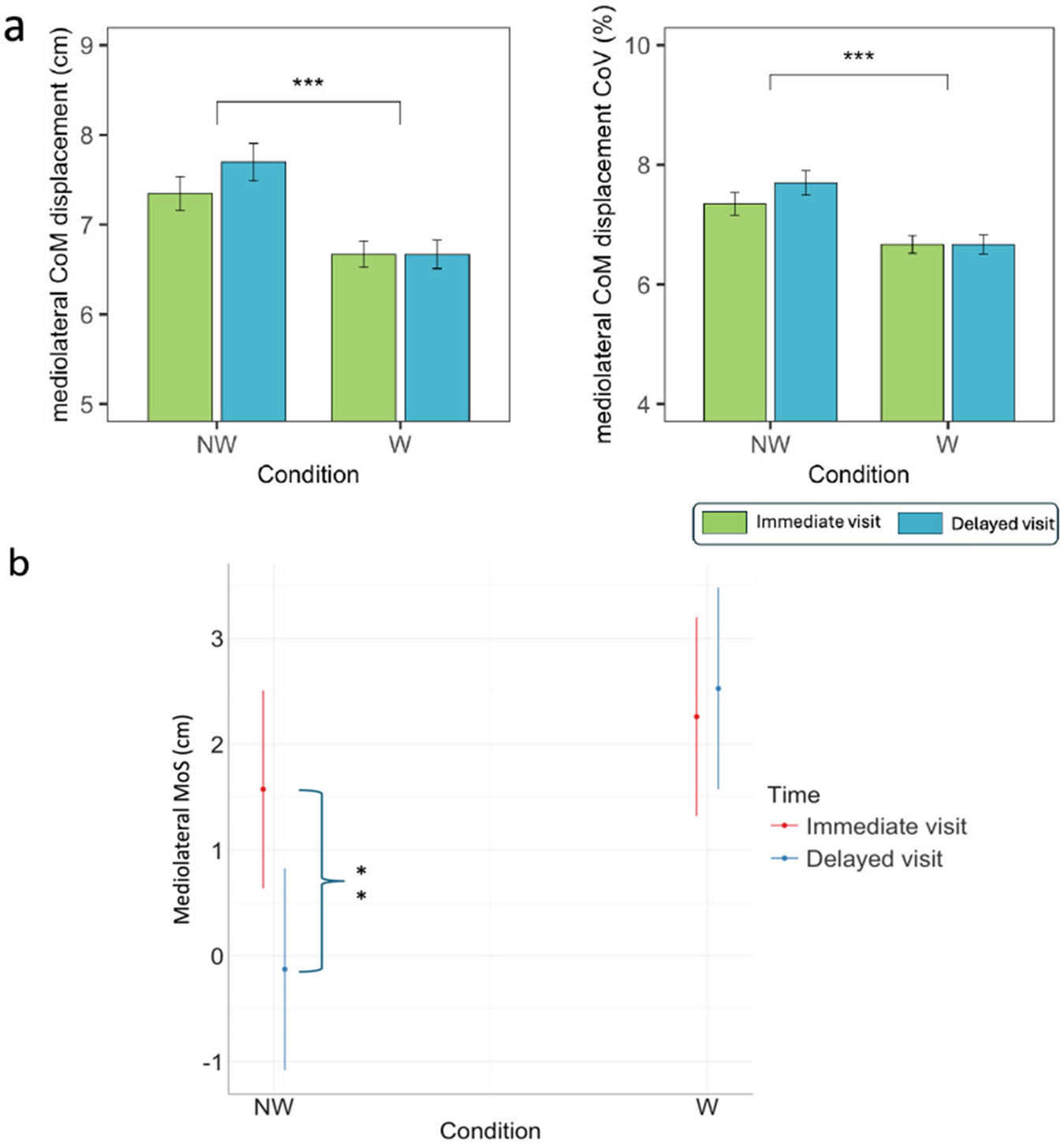
(a) Mediolateral center of mass (CoM) displacement and its coefficient of variation (CoV) for the no-wall (NW) and wall (W) conditions in both the immediate and delayed visit. (b) Interaction plot of mediolateral margin of stability for NW and W conditions in both the immediate and delayed visits. * symbols indicate a significant difference between conditions (* = *p* < 0.05, ** = *p* < 0.01, * * * = *p* < 0.001). Values are reported as mean ± one standard error.

Fig. 7 reports the balance parameters in no-wall and wall conditions, during the immediate and delayed visits.

For the mediolateral margin of stability, the interaction effect between condition (no-wall and wall) and repetition (immediate and delayed visits) was statistically significant (p = 0.003). Pairwise analysis showed that the margin of stability decreased significantly from the immediate visit to the delayed visit in the no-wall condition (*p* = 0.002), while significant changes were not found in the wall condition (*p* = 1.000).

No interaction between condition and repetition was found for the mediolateral CoM displacement. The mediolateral CoM displacement and its variability were significantly lower in wall condition (*p* < 0.001 and *p* < 0.001).

## IV. Discussion

In this study, we investigated gait and balance parameters in the wall and no-wall condition and examined their association with cognitive function while navigating a VR-FMT in vista (no-wall) and environmental (wall) space [32]. Our results showed that in the wall condition, subjects were characterized by smaller, slower, and more variable strides with less body sway. The association analysis demonstrated that in VR-FMT, both with and without walls, the gait and balance parameters were associated with cognitive scores measuring attention and executive function. Additionally, in the wall condition, the gait parameters showed an association with the cognitive scores in the memory domain. These results support the use of gait and balance parameters during navigation as new performance metrics of the floor maze test.

### A. Association Between Cognitive Scores and Maze Completion Time

In the no-wall condition mazes, for the immediate visit, subjects with a longer maze completion time showed a higher Trail Making Test A time. For the delayed visit, subjects with a higher maze completion time showed a higher time in both Trail Making Test A and B. These findings align with results from a prior longitudinal study with the Floor Maze Test (FMT), which demonstrated that higher FMT completion time was associated with decreased performance in Trail Making Test A but not in Trail Making Test B [6]. On the other hand, in the wall condition mazes, for the immediate visit, subjects with a higher maze completion time exhibited a higher Trail Making Test B time and a lower score in the Digit Symbol-Substitution Test and Digit Comparison Test. For the delayed visit, subjects with a higher maze completion time showed a higher Trail Making Test B time and a lower score in the Digit Symbol-Substitution Test. Navigating mazes with walls is a more complex task that challenges multiple cognitive processes evaluated by tests such as Trail Making Test B, Digit Symbol-Substitution Test, and Digit Comparison Test [2].

Our results indicated that the completion time in mazes, both with and without walls, did not show an association with the scores of the memory tests across immediate and delayed visits. During the experiment, we observed that many subjects lost their way while traversing and tried all possible paths to get to the exit point. When subjects got lost in the maze, completion time depended on the speed of executing the path and the randomness of finding the goal rather than the memory capability. This high variance in the maze completion time could confound the potential links between completion time and scores on the memory test.

### B. Gait and Balance Parameters in the Wall Condition and No-Wall Condition

Comparing the no-wall and wall conditions, we observed that subjects in the wall condition exhibited smaller, slower, and more variable strides. This suggests that the wall condition imposes greater cognitive demands, leading to more significant compensatory adaptations in gait [33]. Given that navigation in real world environments, such as buildings, neighborhoods, or towns, cannot be experienced from a single viewing point [14], the gait parameters observed in the wall condition may more accurately reflect those in real-world navigation, providing a better assessment of navigation abilities.

Our results demonstrated that subjects exhibited reduced mediolateral center of mass (CoM) displacement in the wall condition compared to the no-wall condition. This observation aligns with findings from a previous study indicating that increased cognitive load leads to decreased body sway while standing on a platform [34]. This phenomenon may be explained by the hypothesis that cognitive stress prompts individuals to alter their balance by reducing body sway [35]. In the no-wall condition, subjects displayed a significantly lower mediolateral margin of safety (MoS) during the immediate visit than in the delayed visit. This difference was not observed in the wall condition. Learning the no-wall environment might be easier for subjects, allowing them to perform faster gait and compromise balance in the later visit [36]. In contrast, in the wall condition, subjects likely continued to experience cognitive loading, prioritizing maintenance of balance.

### C. Association Between Cognitive Scores and Spatial-Temporal Measures of Gait and Balance in the No-Wall Condition

In the no-wall condition mazes, for the immediate visit, subjects with a higher Trail Making Test A time exhibited reduced stride length and velocity, along with a higher stride time CoV. A higher Trail Making Test B time was linked with a higher stride time CoV. A higher score in the Digit Comparison Test was linked with higher stride velocity. For the delayed visit, subjects with a higher Trail Making Test A time showed a lower stride velocity and higher stride velocity CoV. Subjects with a higher Trail Making Test B time showed a lower stride velocity. Subjects with a higher score in the Digit Comparison Test showed higher center of mass mediolateral displacement CoV. These results demonstrate that subjects with better attention and executive function showed a faster pace with less gait variability during navigation. To complete the maze navigation, subjects had to actively control their walking movement, e.g., slow down and look around when unsure about the planned path and speed up when executing the path. As a result, increased pace and decreased gait variability may be explained by a higher certainty during navigation [37]. Taken together, the results of this study suggest that gait and balance during navigating the VR-FMT without walls correlate with the scores on the cognitive function in the attention and executive function domains.

### D. Association Between Cognitive Scores and Spatial-Temporal Measures of Gait and Balance in the Wall Condition

In wall condition mazes, for the immediate visit, subjects with a higher Trail Making Test A time showed a lower stride length and stride velocity. Subjects with a higher Trail Making Test B time exhibited a lower stride velocity. Subjects with a higher Digit Comparison Test score showed a higher stride velocity and a lower stance phase percentage. For the delayed visit, subjects with a higher Trail Making Test A time showed a lower stride length and stride velocity. Subjects with a higher Trail Making Test B time showed a lower stride length and velocity. A higher Digit Symbol-Substitution Test score was linked with a higher stride velocity. A higher Digit Comparison Test score was linked to a higher stride length and stride velocity and a lower stance phase percentage. Compared to measures in mazes without walls, gait and balance measures in mazes with walls demonstrated a stronger association with executive function, and to a lesser extent attention, which also supports the involvement of more complex cognitive functions in the wall condition. In addition, as hypothesized, measures in mazes with walls were associated with scores in the auditory memory domain. Only in the mazes with walls, subjects need to localize their current position by aggregating information from previously traversed paths into their current path [14]. These results showed that gait parameters in mazes with walls could identify cognitive capability in the memory domain.

### E. Study Limitations and Future Directions

Previous studies have suggested that visual memory plays an important role during navigation [38]. However, our results showed that measures in the VR-FMT were not associated with visual memory scores measured by the Designs Test. In a previous study, Pinjala found that the scores of the Designs Test were not associated with visual memory measured by other tests, and suggested the scores of the Designs Test may not be ideal for the measurement of visual memory [39]. The absence of this association may be due to the fact that the results of the Designs Test were confounded by other aspects of cognitive functioning, such as attention and speed of processing. Future studies should incorporate other visual memory tests such as the visual reproduction test from the Wechsler Memory Scale Edition IV [WMS-IV] [40], Osterrieth Complex Figure Test [41], and Benton Visual Retention Test [42], to further examine the association between spatial navigation ability and visual memory.

Visuospatial reasoning ability measured by the Porteus maze test only presents an association with the center of mass mediolateral coefficient of variation in the wall condition delayed visit. Our results corroborate previous findings that paper-and-pencil spatial tests do not correlate with real-world tests of navigational ability and recruit different brain regions [43]. In comparison with Porteus maze test, active navigation in VR-FMT additionally requires the integration of sensory signals from vestibular, proprioceptive, and podokinetic feedback [9]. Another important feature of active navigation is that subjects divide attention between the cognitive task and motor task during navigation [44], [45]. The continuous interplay between the cognitive task and motor task makes active navigation more difficult to perform than passive navigation, especially in individuals with dementia [10], [46]. Our results supports the idea that navigation tests in an ecological setting are irreplaceable, especially when attempting to predict an individual’s ability to function in their everyday environment [47].

The purpose of the delayed visit design was to enhance the navigation ability evaluation by characterizing the subjects’ spatial memory after a delayed period [48]. For instance, individuals with Alzheimer’s disease rapidly forget what they have heard or saw and are unable to recall after a delay [49]. However, unlike other cognitive tests such as the Logical Memory Test, subjects in the navigation task actively learned the navigational environment during the immediate visit. Consequently, the subjects’ ability to store and retrieve spatial information might be affected by the time a subject spent during the first visit. This was also noted in a previous longitudinal study with FMT, where researchers reported that the performance in the immediate segment was a stronger predictor of predementia syndromes compared to the delayed visit [2]. To further evaluate the spatial memory ability over time, future studies should consider constraining the time for the immediate visit to investigate the spatial memory over time.

During overground walking, steps could be classified into straight steps, turn steps, and spin steps [25]. Each step type is characterized by distinct biomechanical features. For example, during turn and spin steps, the center of mass shifts laterally, in contrast to straight steps, where the center of mass follows a natural mediolateral oscillation between feet placements. In addition, turning is associated with new routes coming into view and decision making, which require additional cognitive resources [50]. However, in this study, only straight walking steps were analyzed as each subject only completed one maze in each condition and the turning data was limited. In our previous work, we have established the method of classifying and analyzing the steps [26]. Future studies should employ more mazes and collect turning data to investigate the association between gait and balance parameters during turn and spin steps to characterize interrelation between navigation and cognitive function.

A longitudinal study is needed to evaluate the potential of using gait and balance performance during VR-FMT to predict future risk of developing dementia syndromes or cognitive decline in nondemented older adults. Specifically, the longitudinal study will examine (i) whether gait and balance parameters during the VR-FMT are associated with progression to dementia, and (ii) the associations between these parameters and established dementia biomarkers.

## V. Conclusion

The current study used VR-FMT to examine the association between gait and balance parameters during spatial navigation and cognitive function, and how this association was affected by the presence of walls in virtual reality. Our results demonstrated that in VR-FMT without walls, gait and balance parameters during navigation were associated with cognitive processes such as attention, executive function. In the VR-FMT with walls, gait and balance parameters were additionally associated with cognitive scores in the auditory memory domain. Our findings suggest that gait parameters could be valid evaluation metrics for spatial navigation. Moreover, VR-FMT with walls could be used in the identification of early impairments in the memory domain.

## Figures and Tables

**TABLE I:** Neuropsychological Test Performance

Domain	Test Variable (units)	Mean ± SD
Attention	Trail Making Test A (time)	37.7 ± 13.4
Executive Function	Trail Making Test B (time)	83.9 ± 41.2
	Digit Symbol-Substitution Test (score, range 0-93)	49.7 ± 13.5
	Digit Comparison (score, range 0-192)	59.7 ± 12.1
Verbal	Control Oral Word Association -’F,A,S’ (score, sum of items named in 60 s for each phoneme)	41.6 ± 12.1
	WRAT4 (Word Reading Subset) (score, range 0-70)	49.4 ± 6.4
Auditory Memory	Immediate Logical Memory Test (score, range 0-50)	29.0 ± 7.0
	Delayed Logical Memory Test (score, range 0-50)	23.7 ± 7.3
Visual Memory	Immediate Design Test (score, range 0-120)	61.6 ± 10.4
	Delayed Design Test (score, range 0-120)	50.8 ± 9.4
Visuospatial Reasoning	Porteus Maze (score, range 0-17)	15.5 ± 1.5

1 WRAT4 = World Range Achievement Test Fourth Edition.

**TABLE II:** Regression Coefficients for Parameters on Neuropsychological Test

	Parameter	Attention	Executive function			Auditory memory		Visual memory		Verbal		Spatial
					
		TMT-A	TMT-B	DSST	DC	ILM	DLM	ID	DD	CO	WRAT4	PM
No-wall Immediate Visit	Completion time	**0.950**	0.187	−0.199	−0.448	−0.274	−0.739	0.234	0.208	−0.419	0.717	1.445
	Stride length	**−0.361**	−0.055	0.127	0.230	0.235	0.430	−0.108	−0.020	0.121	−0.352	−1.039
	Stride velocity	**−0.360**	−0.071	0.182	**0.318**	0.201	0.500	−0.128	−0.068	0.170	−0.340	−0.905
	Stride time CoV	**0.099**	**0.035**	0.039	0.005	−0.057	−0.053	0.059	−0.053	0.021	0.049	0.130
No-wall Delayed Visit	Completion time	**0.724**	**0.214**	−0.144	−0.613	0.182	−0.018	−0.236	−0.293	−0.594	0.014	0.918
	Stride velocity	**−0.326**	**−0.129**	0.108	0.259	0.118	0.316	0.006	0.088	0.147	−0.293	−0.196
	Stride velocity CoV	**0.205**	0.043	0.045	−0.069	−0.125	−0.201	−0.064	−0.071	0.032	0.252	0.488
	CoM ML CoV	−0.027	−0.020	0.094	**0.237**	0.038	0.009	−0.098	−0.038	−0.011	−0.084	−0.202
Wall Immediate Visit	Completion time	1.203	**0.447**	**−1.372**	**−1.459**	−1.232	−2.215	0.850	−0.336	−0.649	−0.248	1.877
	Stride length	**−0.291**	−0.104	0.012	0.291	0.225	0.385	−0.065	0.227	−0.067	−0.222	0.614
	Stride velocity	**−0.327**	**−0.145**	0.133	**0.361**	0.218	**0.476**	0.069	0.240	0.066	−0.186	0.419
	STP	0.065	0.036	−0.065	**−0.104**	0.029	−0.012	−0.033	−0.017	−0.061	−0.033	0.007
	Stride length CoV	0.038	0.004	0.001	−0.003	−0.190	**−0.279**	0.038	−0.001	0.072	0.126	0.358
	Stride time CoV	0.032	0.005	0.041	−0.032	−0.094	**−0.109**	0.050	0.005	0.041	0.057	0.224
	Stride velocity CoV	0.098	0.013	−0.028	−0.016	−0.182	**−0.306**	−0.040	−0.073	−0.025	0.081	−0.207
Wall Delayed Visit	Completion time	1.019	**0.375**	**−1.167**	−1.043	−1.020	−1.700	0.688	−0.299	−0.899	0.504	1.363
	Stride length	**−0.412**	**−0.159**	0.325	**0.408**	0.54	**0.669**	0.021	0.228	0.108	0.035	0.086
	Stride velocity	**−0.384**	**−0.1608**	**0.370**	**0.448**	0.47	**0.644**	0.099	0.240	0.199	0.164	−0.166
	STP	0.037	0.014	−0.036	**−0.082**	−0.016	−0.035	−0.015	−0.023	−0.020	−0.040	0.178
	Stride length CoV	0.034	0.015	−0.035	−0.061	−0.257	**−0.394**	0.174	0.161	−0.009	0.211	0.419
	Stride velocity CoV	0.145	0.045	−0.100	−0.160	−0.345	**−0.509**	0.109	0.078	−0.066	−0.029	0.216
	CoM ML CoV	−0.121	−0.014	−0.034	0.002	0.291	0.318	0.100	−0.093	0.126	−0.005	**−1.777**

1 Abbreviations: TMT-A, TMT-B = Trail Making Test A (time) and Trail Making Test B (time); DSST = Digit Symbol-Substitution Test; DC = Digit Comparison; ILM: Immediate Logical Memory Test; DLM: Delayed Logical Memory Test; ID: Immediate Design Test; DD: Delayed Design Test; CO = Control Oral; WRAT4: World Range Achievement Test (Word Reading Subset); PM: Porteus Maze. CoV: Coefficient of Variation (ratio of the standard deviation to the mean); STP: Stance phase percentage; CoM: Center of Mass; MoS: Margin of Stability.

2 Coefficients are adjusted for demographic variables including age, gender, height, weight, and education. The significant level is set to 0.05. When the association is statistically significant (*p* < 0.05), it is highlighted in bold.
